# Novel Peptide Marker Corresponding to Salivary Protein gSG6 Potentially Identifies Exposure to *Anopheles* Bites

**DOI:** 10.1371/journal.pone.0002472

**Published:** 2008-06-25

**Authors:** Anne Poinsignon, Sylvie Cornelie, Montserrat Mestres-Simon, Alessandra Lanfrancotti, Marie Rossignol, Denis Boulanger, Badara Cisse, Cheikh Sokhna, Bruno Arcà, François Simondon, Franck Remoue

**Affiliations:** 1 UR024-Epidémiologie et Prévention, IRD (Institut de Recherche pour le Développement), Dakar, Sénégal; 2 UR024-Epidémiologie et Prévention, IRD, Montpellier, France; 3 Department of Public Health, Parasitology Section, Sapienza University, Rome, Italy; 4 UR077-Paludologie Afro-tropicale, Dakar, Sénégal; 5 Département de Parasitologie, Université Cheikh Anta Diop, Dakar, Sénégal; 6 Department of Structural and Functional Biology, University Federico II, Naples, Italy; Centre for DNA Fingerprinting and Diagnostics, India

## Abstract

**Background:**

In order to improve malaria control, and under the aegis of WHO recommendations, many efforts are being devoted to developing new tools for identifying geographic areas with high risk of parasite transmission. Evaluation of the human antibody response to arthropod salivary proteins could be an epidemiological indicator of exposure to vector bites, and therefore to risk of pathogen transmission. In the case of malaria, which is transmitted only by anopheline mosquitoes, maximal specificity could be achieved through identification of immunogenic proteins specific to the *Anopheles* genus. The objective of the present study was to determine whether the IgG response to the *Anopheles gambiae* gSG6 protein, from its recombinant form to derived synthetic peptides, could be an immunological marker of exposure specific to *Anopheles gambiae* bites.

**Methodology/Principal Findings:**

Specific IgG antibodies to recombinant gSG6 protein were observed in children living in a Senegalese area exposed to malaria. With the objective of optimizing *Anopheles* specificity and reproducibility, we designed five gSG6-based peptide sequences using a bioinformatic approach, taking into consideration i) their potential antigenic properties and ii) the absence of cross-reactivity with protein sequences of other arthropods/organisms. The specific anti-peptide IgG antibody response was evaluated in exposed children. The five gSG6 peptides showed differing antigenic properties, with gSG6-P1 and gSG6-P2 exhibiting the highest antigenicity. However, a significant increase in the specific IgG response during the rainy season and a positive association between the IgG level and the level of exposure to *Anopheles gambiae* bites was significant only for gSG6-P1.

**Conclusions/Significance:**

This step-by-step approach suggests that gSG6-P1 could be an optimal candidate marker for evaluating exposure to *Anopheles gambiae* bites. This marker could be employed as a geographic indicator, like remote sensing techniques, for mapping the risk of malaria. It could also represent a direct criterion of efficacy in evaluation of vector control strategies.

## Introduction

The threat from vector-borne diseases, considered to be major public health problems in developing countries, is prompting research and health community in developing new tools for diseases control. Malaria is by far the most severe of these diseases. It is transmitted by the *Anopheles* mosquitoes and is responsible each year for at least 400 million acute cases globally, resulting in more than one and a half million deaths [1]. The vast majority of malaria deaths occur in sub-Saharan Africa and are caused by *Plasmodium falciparum,* the most severe and life-threatening form of the disease. In these areas the *Anopheles gambiae* complex is the major vector. With a goal toward improving malaria control, the “*Roll Back Malaria*” partnership has recommended developing new diagnostic tool, especially for identifying geographic areas with high risk of transmission [1]. The evaluation of exposure to malaria risk is currently based on entomological methods (traps, household/indoor spraying, human-landing catches, etc.) but such methods are mainly applicable at the population level and do not enable evaluation of the heterogeneity of individual exposure. Trapping methods using adult volunteers can estimate individual exposure, but may be limited due to ethical constraints and limitations in terms of extrapolation to the incidence in children [2].

*Plasmodium* parasites are injected together with saliva during blood-feeding by an infected *Anopheles* female. Salivary proteins play a dual role in facilitating mosquito blood feeding; their pharmacological properties permit to counteract human defenses triggered by dermis disruption (inflammatory and hemostasis) and their immunological properties modulate the immune response of the human host [3], [4]. In addition, some salivary proteins are immunogenic and can initiate a specific antibody (Ab) response [5]. Linked to this interesting property, previous studies have shown that the anti-saliva Ab response could be a potential marker of exposure to vector-borne diseases in individuals exposed to bites of arthropod vectors, such as ticks [6], phlebotomies [7], *Triatoma* [8], *Glossina* [9] and also *Aedes* mosquitoes [10]. As concerns *Anopheles spp*. and malaria transmission, early epidemiological studies have shown that individuals living in malaria endemic areas, i.e. exposed to *Anopheles* bites, develop a specific anti-saliva Ab response [11], [12]. In Senegal, our group has indeed demonstrated that the IgG response to whole saliva extracts (WSE) of *An. gambiae* represents a marker of exposure to *An. gambiae* bites. In addition, high anti-saliva IgG levels appeared to be a predictive indicator of malaria morbidity [11].

Some families of salivary proteins are widely distributed in bloodsucking *Diptera* [13]. Taking this into account, the evaluation of *Anopheles* exposure based on the immunogenicity of WSE could be skewed and/or overestimated by possible cross-reactivity between common epitopes on immunogenic salivary proteins between mosquito species. An alternative for optimizing the specificity of this immuno-epidemiological test would thus be to identify *Anopheles* genus-specific proteins [14].

In the last decade, biochemical properties and the role played by saliva and salivary glands of arthropods in the permissiveness of transmission of pathogens has become a new research pathway for disease vectors [15], [16]. Related to the identification of the arthropod genome, these studies were performed by high throughput transcriptome and proteome analyses based on salivary gland cDNA libraries [17]. In *An. gambiae*, a catalogue including 71 secreted salivary proteins has recently been described [18]. Among these proteins, the so-called Salivary Gland proteins (SG1-8) have been reported to be *Anopheles spp.*-specific and may represent potential candidate for elaborating genus-specific markers of exposure [19].

Furthermore, among these specific proteins, recent data indicated that the gSG6 protein could be immunogenic in individuals exposed to *Anopheles*. Indeed, via an immunoblotting approach, a band with molecular weight corresponding to the gSG6 protein (11–13 kDa) was identified as antigenic in individuals briefly exposed to *Anopheles* bites [20]. In addition, in Senegalese children living in an endemic area for malaria, the gSG6 protein was recently confirmed as being antigenic by a 2D approach coupled with mass spectrometry (Cornelie, unpublished data). Above these 2 criteria, the gSG6 protein would seem to be a relevant candidate for validating its potential as an immunological marker specific to *An. gambiae* bites.

The objective of the present study was to determine whether the IgG Ab response to the *An. gambiae* gSG6 antigen and derived peptides is an immuno-epidemiological marker of exposure specific to *An. gambiae* bites in children living in an endemic area for malaria. Using a step-by-step approach, we investigated i) the antigenicity of gSG6 expressed in recombinant form, ii) the *Anopheles*-specificity and the antigenic potential of gSG6 peptides designed using a bioinformatic approach, and iii) anti-gSG6 peptides IgG levels in exposed children according to *An. gambiae* exposure as estimated by entomological methods.

## Materials and Methods

### Study population

The present study was conducted in Niakhar, a rural district of central Senegal. This area is characterized by a dry savannah with a rainy season from July to October (approximately 400 mm of rainfall recorded). This area is typical of the Sahel and Sub-Sahel regions of Africa, where the occurrence of malaria is unstable, with a season of *P. falciparum* transmission mainly from September to November [21], [22]

Sera were available from a clinical trial on seasonal intermittent preventive treatment for prevention of malaria performed in 2002 in children aged 6 weeks to 5 years [22]. Sera from a subsample of these children were available at the peak (September) and at the end (December) of the rainy season, as previously described [11]; 241 children were screened in September 2002 and, among them, 175 children were screened in both September and December 2002.

Both the trial on anti-malaria treatment and the present study followed ethical principles according to the Edinburgh revision of the Helsinki Declaration, and were approved by the ethical committees of the Ministry of Health of Senegal (August 2002 and May 2003, respectively) and of the IRD (Institute of Research for the Development) (January 2004). The anti-malaria trial was approved by the ethical committee of the London School of Hygiene and Tropical Medicine in June 2002. Written informed consent was obtained from the study population.

### Entomological data

Entomological data were collected each month between September and December 2002 in 11 villages in the Niakhar area using capture by light traps (CDC miniature light trap). Analysis of entomological data led to defining 3 groups of individuals classified by their exposure level to *Anopheles gambiae* bites (low, medium and high exposure levels) as previously described [11].

### Recombinant protein gSG6

*An. gambiae* gSG6 was expressed as recombinant protein in the yeast *Pichia pastoris* (Arcà B., unpublished data). cDNA coding for mature gSG6 polypeptide was amplified by RT-PCR using gene-specific oligonucleotide primers. Amplified fragments were directionally cloned in the pPICZα vector (Invitrogen, Carlsbad, CA) between sequences coding for the signal peptide of the *Saccharomyces cerevisiae* α factor at the N-terminus and those coding for the *c-myc* and polyhistidine tags at the C-terminus. Protein expression was induced by addition of methanol (0.5%) to the medium. After 24–48 hours, supernatants were collected by centrifugation and the proteins were purified by Ni-NTA affinity chromatography according to the manufacturer's instructions (Quiagen, Ontario, Canada). Affinity-purified fractions were employed for the determination of IgG levels by ELISA.

### Peptide design of gSG6 salivary protein

We investigated the design of potential immunogenic peptides of the gSG6 protein using bio-informatic tools. The strategy was i) to identify potential immunogenic epitopes predicted by algorithms; and ii) to research the specificity of *An. gambiae* gSG6 peptide sequence compared to the genome/ Expressed Sequence Tag (EST) libraries of other organisms.

This analysis was based on the amino acid sequence of mature *An. gambiae* gSG6 (“UniProtKB/TrEMBL:Q9BIH5” and “gi:13537666”, [23]).

The identification of putative linear B-cell epitopes of *An. gambiae* gSG6 was performed by computerized predictions of antigenicity based on physico-chemical properties of the amino-acid sequences with the BcePred database [24] and with the FIMM database [25]. We also identified the MHC class 2 binding regions using the ProPred-2 online service [26].

Sequence alignments were done with the Tblastn program in Vectorbase database [27] which enabled comparing a sequence of gSG6 peptides with known genomes or EST libraries of *Aedes aegypti, Ixodes scapularis, Culex pipiens, Pediculus humanus, Glossina morsitans, Rhodnius prolixus, Lutzomia longipalpis* and *Phlebotomus papatasi*. Concomitantly, we investigated sequence alignments with the Blast program to compare the gSG6 peptides sequence with all non-redundant GenBank CDS database [28].

Peptides were synthesized and purified (>80%) with Genosys (Sigma-Genosys, Cambridge, UK) with an added N-terminal biotin. All peptides were shipped lyophilized and they were resuspended in 0.22 µm filtered milliQ water and stored in aliquots at −80°C.

### Evaluation of human IgG Ab levels ELISA

ELISA was carried out using salivary antigens (gSG6 recombinant protein or biotinylated gSG6 peptides) and sera were tested for IgG antibodies. Maxisorp plates (Nunc, Roskilde, Denmark) were coated with recombinant protein (5 µg/mL) or gSG6 peptides (20 µg/mL for gSG6-P1, gSG6-P5 and 30 µg/mL for gSG6-P2, gSG6-P3, gSG6-P4) in carbonate/bicarbonate buffer. Individual sera were incubated in PBS-Tween 1% (1:10 for assessment on recombinant protein, gSG6-P2 (subsample n<30) and gSG6-P3, and 1:20 for assessment on gSG6-P1, gSG6-P2 (large sample n = 241), gSG6-P4 and gSG6-P5). Anti-gSG6 peptides IgG detection was performed using an HRP goat anti-human IgG Ab (1:25000, Nordic Immunology, Tilburg, Netherlands) and anti-recombinant protein IgG detection was performed using a mouse biotinylated mAb (1:1000, BD Pharmingen, San Diego, CA). Peroxidase-conjugated streptavidin (1:1000, Amersham, Les Ulis, France) was added only for assay using biotinylated secondary antibodies. Colorimetric development was carried out using ABTS (2,2′-azino-bis (3-ethylbenzthiazoline 6-sulfonic acid) diammonium; Sigma, St Louis, MO) in 50 mM citrate buffer (pH 4) containing 0.003% H_2_O_2_. Absorbance/Optical Density (OD) was measured at 405 nm. In addition, the absence of significant Ab detection was verified in wells without antigen (ODn). Individual results were expressed as ΔOD value calculated according to the formula ΔOD = ODx-ODn, where ODx represented the individual OD value in antigen wells.

### Statistical analysis

All data were analyzed with GraphPad Prism software® (San Diego,CA, USA). After verifying that values did not assume Gaussian distribution, the non-parametric Mann-Whitney U-test was used for comparison of Ab levels between two independent groups and the non-parametric Kruskal-Wallis test was used for comparison between more than two groups. The Wilcoxon matched pair test was used to compare paired sera between September and December. All differences were considered significant at P<0.05.

## Results

### Immunogenicity of gSG6 recombinant protein

The IgG response to recombinant gSG6 protein was evaluated in a small sample of children living in the studied area. The children (n = 16) chosen for this initial test were selected for their high level of IgG Ab specific to whole *An. gambiae* saliva (0.532<OD_WSE_<1.499, [11]).

**Figure 1:**
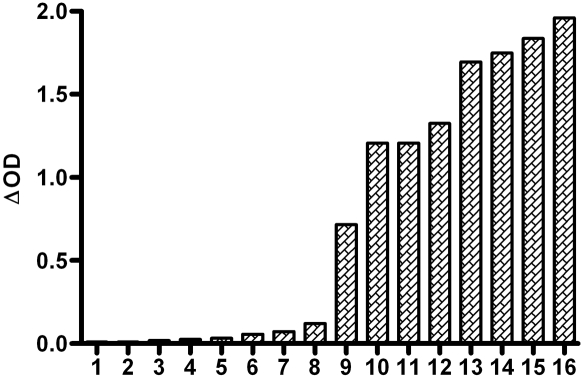
IgG antibody response specific to gSG6 recombinant protein. The IgG antibody level was evaluated in children (n = 16) living in an endemic area for malaria. Individual ΔOD results (ΔOD as described in the “Materials and Methods” section) at the peak of the season of *Anopheles* exposure (September) are reported. Samples are ordered according to the intensity of the individual ΔOD value.

The level of IgG Ab to gSG6 (0.01<ΔOD<1.959) classified according to the intensity of the ΔOD value is presented in Figure 1. A high anti-gSG6 IgG response was observed in half of the children. Interestingly, important variations in the anti-gSG6 IgG level were observed between exposed individuals ranging from a low (Ind. 1–8) or intermediate (Ind. 9) to a high intensity of the Ab level (Ind. 10–15).

### Peptide design

The second step was to design gSG6-based peptides with the objective of optimizing and increasing *Anopheles* specificity and reproducibility of the assay, and overcoming limits in production of the recombinant protein and possible batch-to-batch variations.

**Figure 2:**
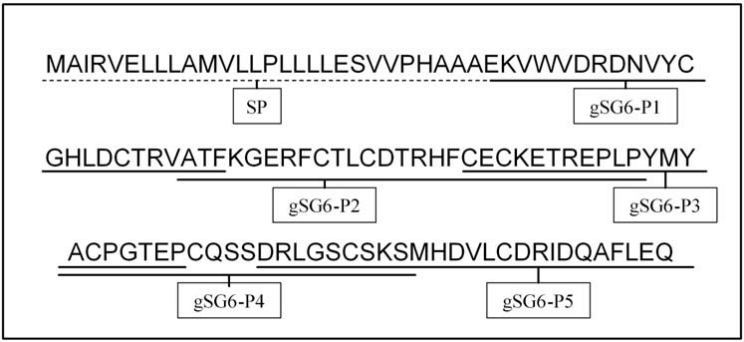
Amino-acid sequence of gSG6 Peptides. Amino-acid sequence of the SG6 protein of *Anopheles gambiae* (gi:13537666) is presented and sequences of the selected peptides, gSG6-P1 to gSG6-P5, are underlined. Signal peptide (SP) sequence is indicating by dotted underline.

The identification of potential immunogenic epitopes of the gSG6 protein was done with bioinformatic tools. We employed several algorithms for prediction of potential immunogenic sites (putative linear B-cell epitopes and MHC class 2 binding regions). The crossing of the immunogenic predicted epitopes led us to define 5 gSG6 peptides (gSG6-P1 to gSG6-P5) of 20 to 27 amino acid residues in length, overlapping by at least 3 residues and spanning the entire sequence of the mature gSG6 protein. Both predictive methods for putative linear B-cell epitopes (FIMM and BcePred) assigned the highest potential immunogenicity to gSG6-P1. This peptide was followed in the predicted immunogenicity scale by gSG6-P2 (according to BcePred) or by gSG6-P3 and gSG6-P4 (according to FIMM). Peptide sequences are shown in Figure 2.

In order to try to maximize *Anopheles* specificity and to avoid potential immune cross-reactivity (with proteins from other vector species as well as from pathogens or other organisms), we also searched for similarities using the Blast family programs, including both the genome/EST libraries of other vector arthropods available in Vectorbase and of pathogens/organisms in non-redundant GenBank CDS databases. No relevant similarity was found with proteins of other bloodsucking arthropods, as indicated by the low scores that were obtained (few amino acids consecutively matched and high rate of e-value, i.e. e>0.13). Indeed, the longest perfect match was six amino acids between a putative protein from *Pediculus humanus* and gSG6-P2 and gSG6-P3 peptides (e = 0.56). In the case of gSG6-P1, the best match was four amino acids in length with *Culex pipiens quinquefasciatus* salivary adenosine deaminase (e = 0.95). Moreover, no relevant similarity was found with sequences from pathogens or other organisms. The highest hits of gSG6-P1 were with the cyanobacterium *Microcystis aeruginosa* (three amino acids, e = 2.6) and with *Ostreococcus* OsV5 virus (four amino acids, e = 15).

This analysis confirmed the *bona fide* high specificity of the 5 selected gSG6 peptides for the *Anopheles* species.

### Immunogenicity of gSG6 peptides

**Figure 3:**
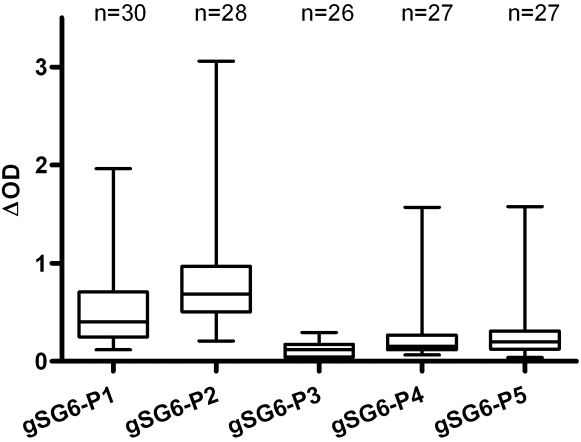
IgG antibody response according to gSG6 peptides. For each gSG6 peptide, the IgG antibody level was evaluated in a subsample of children living in the studied area. Results at the peak of the season of *Anopheles* exposure are reported according to gSG6 peptides. Results are presented by box plot graph where lines of the boxes represent the 75th percentile, median and 25th percentile of individual average ΔOD values; whiskers represent the lower and upper adjacent values.

The following step was carried out to evaluate the IgG Ab response to the five gSG6 peptides by ELISA in a randomly selected subsample of children (n<30) living in the studied area (Figure 3). All peptides were antigenic, but the intensity of the IgG level was clearly peptide-dependent; weak antigenicity was observed for gSG6-P3, gSG6-P4 and gSG6-P5, whereas gSG6-P1 and gSG6-P2 appeared highly antigenic in this subsample of children.

### IgG response to gSG6 peptides according to exposure to *An. gambiae* bites

**Figure 4:**
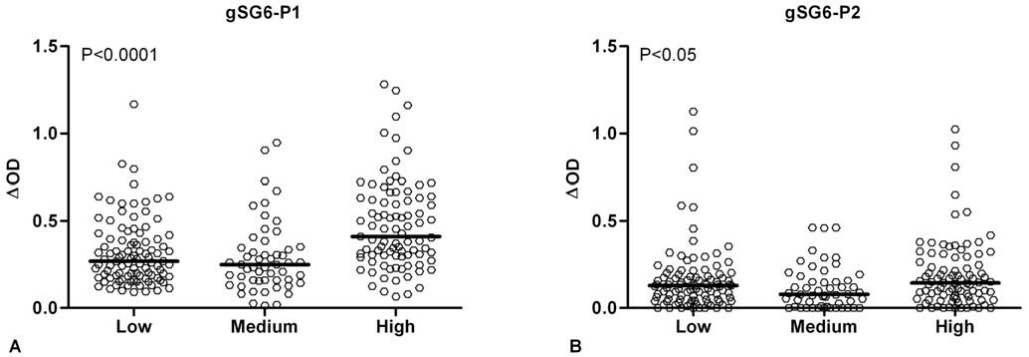
IgG response to gSG6-P1 and gSG6-P2 according to intensity of exposure to *Anopheles gambiae* bites. Individual ΔOD values in September (peak of the season of *Anopheles* exposure) are shown for the three different exposure groups. Results are presented for the same children (n = 241) for gSG6-P1 (A) and gSG6-P2 (B). Exposure groups were defined by entomological data. Bars indicate median value for each exposure group. Statistical significance between the 3 groups is indicated (non-parametric Mann-Whitney U-test).

Entomological data led to defining 3 groups of individuals classified by their exposure level (low:1.29±1.11; medium:16.75±3.18; high:31.08±3.47 bites/human/night, mean±SD) as previously described [11]. Therefore, for each gSG6 peptide, we first compared the specific IgG level according to their exposure group, within the same randomly selected subsample of children (data not shown). A positive trend between the specific IgG level and the intensity of exposure was found for gSG6-P1 and for gSG6-P2 in this subsample and this association was only significant for gSG6-P1 (P<0.05). In contrast, gSG6-P3, gSG6-P4 and gSG6-P5 were weakly immunogenic, as also previously mentioned (Figure 3), In addition, the intensity of IgG Ab levels to these 3 peptides was similar whatever the 3 groups of exposure level (data not shown). For all these reasons, the next stage consisted of validating only gSG6-P1 and gSG6-P2 peptides as markers of exposure in a larger immuno-epidemiological analysis according to entomological data. The IgG level specific to gSG6-P1 and gSG6-P2 was then evaluated in children (n = 241) according to their exposure group at the peak of the season of *An. gambiae* exposure (September), as defined by entomological data (Figure 4). The IgG response showed significant differences according to exposure groups for both peptides (P<0.0001 for gSG6-P1 and P = 0.0195 for gSG6-P2, respectively, Kruskal-Wallis test). The anti-gSG6-P1 IgG level was similar in children in the low and medium exposure groups, whereas it was significantly higher in children from the high exposure group (P<0.0001 versus both low and medium groups). In contrast, the median anti-gSG6-P2 IgG level appeared closely similar between the low and high groups of exposure (non-significant), although a significant difference (P<0.05) was observed when comparing medium and high groups. In addition, the evolution of the specific IgG antibody response to gSG6-P1 and gSG6-P2 peptides in children (n = 175) was evaluated between the peak (September) and the end (December) of the exposure season (data not shown). The specific IgG response was significantly higher in December as compared to September only for gSG6-P1 (P = 0.0137, Wilcoxon matched pairs test). Altogether, these results showed that only the IgG response to gSG6-P1 increased with the level of exposure to *An. gambiae*, evaluated by classical entomological data.

## Discussion

In this present study, the Ab response specific to *Anopheles gambiae* gSG6 salivary protein, from its recombinant form to synthetic peptides, was investigated in children living in a malaria endemic area in Senegal. We have shown for the first time that exposed individuals develop an IgG response to gSG6 protein, with considerable variations among children. With the objective of optimizing *Anopheles* specificity and reproducibility of the immunological assay, a peptide design approach was undertaken using bioinformatic tools. Based on these analyses, five gSG6 peptides were selected for their potential immunogenic properties and for their presumed absence of cross-reactivity (on the basis of identity and similarity) with proteins of other arthropod vectors or pathogens/organisms whose genome or EST libraries are available. The specific IgG level to gSG6 peptides was then evaluated according to the level of exposure as estimated by entomological data. The five gSG6 peptides were antigenic, but the intensity of their specific IgG responses appeared peptide-dependent. Indeed, gSG6-P1 and gSG6-P2 showed the highest level of antigenicity in exposed children, whereas gSG6-P3, gSG6-P4 and gSG6-P5 presented lower levels. This immuno-epidemiological analysis confirmed bioinformatic predictions and enabled us to identify gSG6-P1 and gSG6-P2 as high antigenic peptides. However, only the IgG response to gSG6-P1 increased with the degree of exposure to *An. gambiae* bites, as estimated by classical entomological methods, and in agreement with previous observations of the IgG response to WSE in the same area [11]. Along with the significant increase in anti-gSG6-P1 IgG during the rainy season, these results indicated that the IgG Ab response to gSG6-P1 was positively associated with the exposure to the *An. gambiae* vector. Overall, this step-by-step original approach points to gSG6-P1 as a potential candidate as an immuno-epidemiological marker of exposure to *An. gambiae* bites.

The evaluation of immune responses to salivary components might represent a means for assessing individual exposure to vector bites. Previous studies on malaria vectors investigated the Ab response to *Anopheles* WSE or to salivary gland extracts in individuals living in malaria endemic areas. The specific IgG response to *Anopheles* saliva appeared to be a potential indicator of exposure to vector bites in Senegal [11] and Thailand [12]. Nevertheless, collection of saliva and/or salivary gland extracts is tedious and time-consuming; in addition, saliva composition can be affected by several ecological parameters such as age, feeding status or infectivity of *Anopheles* [29], which in turn may influence the anti-saliva immune response measured in exposed individuals. A further complication lies in the widespread occurrence of some families of salivary proteins in hematophagous arthropod species, which may induce the presence of cross-reactive antibodies in human sera [13]. An ideal alternative would be the availability of a single immunogenic salivary protein specific to a given vector species. This approach has been previously investigated by our team for *Glossina* species, vectors of *trypanosomatidae* [30] and recently for *An. gambiae* [31]. To define an immunological marker of specific exposure to *Anopheles* genus, in terms of specificity, sensitivity and reproducibility, we explored the composition in salivary proteins of different insect vector species among transcriptomic and genomic studies. The gSG6 salivary protein, first described in *An. gambiae* [32], was further reported as being specific to *Anopheles* mosquitoes and highly conserved among *Anopheles* species [19], [33]. Indeed, sequence alignment indicated high identity: SG6 of *An. gambiae* shared 75% identity with *An. stephensi* and 76% with *An. funestus* [34]. In addition, the *An. gambiae* gSG6 protein has been reported to be potentially antigenic in travelers exposed for short periods to *Anopheles* bites [20], and it was confirmed as being antigenic in Senegalese children by an immuno-proteomic approach coupling 2D immunoblot and mass spectrometry (Cornelie S., unpublished data). In the present study, we observed that the level of IgG to gSG6 protein was clearly individual-dependent and may represent a tool to discriminate exposure at the individual level. Altogether, these results indicated the strong potential of gSG6 protein as a candidate marker of exposure to *Anopheles gambiae* bites.

The peptide design strategy has strengthened the specificity of markers to *An. gambiae* and in particular, we demonstrated that a single peptide, gSG6-P1 could be the candidate as a marker of exposure. Indeed, this peptide appeared to satisfy several requirements that such an exposure marker should fulfill. First, it thus far appears to be specific to the *Anopheles* genus and therefore, no relevant cross-reactivity phenomena with epitopes from other proteins (main *Diptera* species or pathogens) would be expected. Nevertheless, few vector genomes are currently available and the clear absence of cross-reactivity will need to be confirmed among other major insect vectors. Second, because it is of a synthetic nature, it guarantees high reproducibility of the immunological assay. Third, it elicits a specific Ab response which correlates well with the level of exposure to *An. gambiae* bites.

In the studied area, the main vector of *Plasmodium falciparum* has been reported to be *An. arabiensis* [11], [21], a species belonging to the *An. gambiae s.l.* complex and whose genome is not currently available. gSG6 peptides were designed on the basis of the *An. gambiae s.s.* sequence, the only *Anopheles* genome available [35], which may perhaps result in an under-estimation of the immune response in the studied children, as previously mentioned [11]. Nevertheless, gSG6-P1 shares 82% and 91% identity with *An. stephensi* and *An. funestus*, respectively, two species with greater evolutionary distance from *An. gambiae s.s.* as compared to *An. arabiensis*. The above observation tends to support the notion that gSG6-P1 can be used to evaluate the exposure to other *Anopheles* vectors of malaria. Obviously, confirmation in other transmission areas presenting different malaria transmission modalities is needed. In addition to the applications already mentioned, there exist a number of other useful applications of a marker of exposure to *Anopheles* bites. It will be interest to evaluate gSG6 peptides in areas with different modalities of transmission, both in term of intensity and of the dynamics of exposure. For example, it would be very useful to identify low exposure, for which entomological studies are not sensitive enough (dry season, malaria according to altitude, urban exposure) or adequate (travelers, military corps).

One direct application of such a gSG6 peptide marker of exposure could lie in the elaboration of maps representing the risk of exposure to *Anopheles* bites. The development of such immuno-epidemiological markers might represent a quantitative tool applied to field conditions and a complementary tool to those currently available, such as entomological, ecological and environmental data [36]. It could represent a geographic indicator of the risks of malaria transmission and thus a useful tool for predicting malaria morbidity risk as previously described [5]. Furthermore, it may represent a powerful tool for evaluation of vector control strategies (impregnated bednet, intradomiciliary aspersion, etc.) and could here constitute a direct criterion for effectiveness and appropriate use (malaria control program).

In conclusion, we have developed an original approach coupling bioinformatic and immuno-epidemiological tools, which succeeded in identifying a candidate for developing a marker of exposure to *An. gambiae* bites. A similar methodology could be applied to the challenge inherent in control of other vector-borne diseases.
